# P02.145. Relationship of treatment beliefs to subject blinding: the case of a placebo-controlled RCT in Irritable Bowel Syndrome

**DOI:** 10.1186/1472-6882-12-S1-P201

**Published:** 2012-06-12

**Authors:** M Quilty, L Conboy

**Affiliations:** 1 Harvard School of Public Health, Boston, USA; 2 New England School of Acupuncture, Newton, USA

## Purpose

Subject blinding is critical to minimizing bias in randomized controlled trials (RCTs). Irritable Bowel Syndrome (IBS) is a complex and often chronic condition with physical and psychological components. For many patients, psychological issues such as anxiety and stress can exacerbate the existing condition. Given the mind-body relationship in this illness, we explored if an individual’s belief in treatment and a belief in being randomized to an active treatment group could impact their self-reported health outcomes.

## Methods

In the parent study, data were gathered in a three-week randomized controlled trial (n=262) testing the therapeutic effect of an Augmented (supportive) patient-practitioner relationship versus a Limited patient-practitioner relationship versus wait list in reducing the symptoms of IBS. During the first three weeks of the trial, all treatments were given in the context of biweekly sham acupuncture treatments to allow the effects of patient-practitioner relationship to be isolated. The second three weeks of the trial began with a blind rerandomization of subjects in the two treatment arms to either continue sham acupuncture or begin real (insertive) acupuncture. Symptom data, expectations, and beliefs concerning blinding were collected at baseline, 3, and 6 weeks. Expectations of treatment group assignment (insertive or sham) and symptom improvement were collected after the first treatment and before weeks 3 and 6 data collection.

## Results

For all six of our expectancy and belief variables, the Augmented and insertive treatment groups had more positive reports compared to the Limited and sham acupuncture group, respectively; this trend continued in reported symptom relief as well.

## Conclusion

The risk of subject unblinding may be related to qualities of the patient-practitioner relationship and treatment experience; a better understanding of blinding as a data safeguard is necessary.

